# Sporadic neurofibroma of transverse colon in a patient without neurofibromatosis type 1: A case report

**DOI:** 10.1016/j.ijscr.2020.04.024

**Published:** 2020-05-08

**Authors:** Toru Imagami, Saburo Sugita, Takaya Nagasaki, Masahiro Kimura, Keisuke Ito, Shingo Inaguma

**Affiliations:** a Department of Surgery, Nagoya City East Medical Center, Nagoya, Japan; b Department of Gastroenterology, Nagoya City East Medical Center, Nagoya, Japan; c Department of Pathology, Nagoya City East Medical Center, Nagoya, Japan

**Keywords:** NF1, neurofibromatosis type 1, CT, computed tomography, CME, complete mesocolic excision, MPNSTs, malignant peripheral nerve sheath tumors, Neurofibroma, Colon, Endoscopy, Laparoscopic surgery

## Abstract

**Introduction:**

The occurrence of sporadic colonic neurofibroma particularly in a patient without neurofibromatosis type 1 has been rarely reported. Therefore, the clinical significance of this disease has not been fully elucidated.

**Presentation of case:**

An 81-year-old woman with a positive fecal occult blood test result was referred to our institution for the evaluation of anemia. On colonoscopy, a 50-mm submucosal tumor-like mass was found in the hepatic flexure of the colon. Superficial biopsy and boring biopsy showed unspecific granulation tissues, and immunostaining revealed that the mesenchymal tumor was negative for CD34, c-kit, desmin, and S100 protein. The patient underwent laparoscopic right colectomy with complete mesocolic excision (CME). Pathologically, the tumor was diagnosed as neurofibroma.

**Discussion:**

Gastrointestinal neurofibromas are known to cause clinical symptoms. No colonic neurofibroma has been diagnosed before resection. Moreover, neurofibromas, particularly large lesions, reportedly undergo malignant transformation. Surgical extirpation with clear margins is the primary treatment, and laparoscopic surgery is considered acceptable for colonic neurofibroma and colon cancer.

**Conclusion:**

Based on our experience, a preoperative diagnosis was impossible for colonic neurofibroma. Laparoscopic surgery with CME is considered feasible for sporadic colonic neurofibroma.

## 1 Introduction

Neurofibromas, commonly observed in individuals with neurofibromatosis type 1 (NF1), are benign tumors that arise from Schwann cells owing to a mutation of the tumor suppressor gene *NF1* [1]. In patient with NF1, although as many as 25% of cases involve the gastrointestinal tract, most cases involve the stomach or small bowel and colonic involvement is rarely observed [2]. Previously, only a few cases of sporadic colonic neurofibroma have been reported in patients without NF1 [[1], [2], [3], [4], [5], [6], [7]]. Owing to its rarity, the clinical features of sporadic colonic neurofibroma have not been fully elucidated.

Here we report a rare case of sporadic neurofibroma of the transverse colon in a patient without NF1. With this report, our experience can contribute to a better understanding of colonic neurofibromas. This work has been reported in line with the SCARE criteria [8].

## 2 Presentation of case

**Fig. 1:**
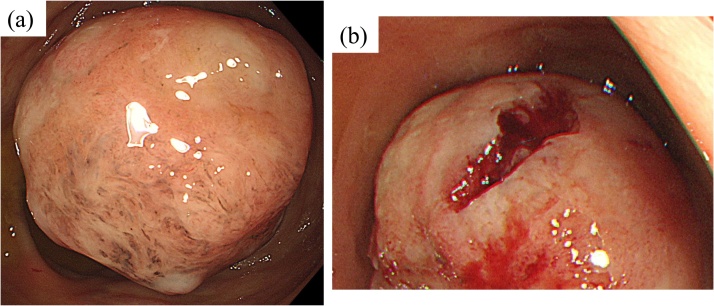
Colonoscopic findings. (a) A 50-mm submucosal tumor-like mass was found in the hepatic flexure of the colon. (b) Superficial biopsy and boring biopsy were performed

**Fig. 2:**
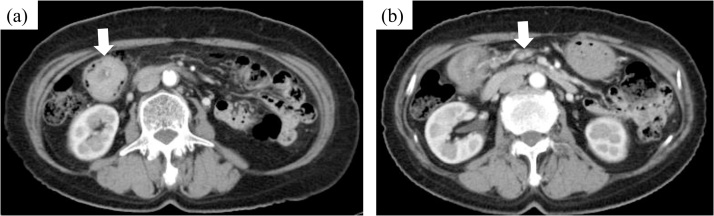
Contrast-enhanced computed tomography findings. (a) The tumor occupied the transverse colon. (b) Several regional lymph nodes were swollen.

An 81-year-old woman with positive fecal occult blood test result was referred to our hospital for the evaluation of anemia. The patient had a medical history of hyperlipidemia but no familial medical history. Laboratory examination revealed iron deficiency anemia (hemoglobin, 7.0 g/dL; iron, 63 μg/dL; white blood cell, 7160/μL; and platelet count, 367000/μL). Moreover, her liver function, renal function, and tumor marker levels were within normal limit. On colonoscopy, a 50-mm submucosal tumor-like mass was found in the hepatic flexure of the colon (Fig. 1). Superficial biopsy and boring biopsy revealed unspecific granulation tissues, and immunostaining revealed that the mesenchymal tumor was negative for CD34, c-kit, desmin, and S100 protein. In addition to the presence of granulation tissues, a high infiltration of lymphocytes and plasma cells was observed. Contrast-enhanced computed tomography (CT) revealed a colonic tumor and swelling regional lymph node in the transverse colon (Fig. 2). Although an accurate diagnosis was not obtained and possibility of malignancy was not confirmed, the tumor was believed to have caused anemia in this case. Therefore, we decided to perform resection for the treatment of colon cancer. After an informed consent was obtained from the patient and her family, she underwent laparoscopic right colectomy with complete mesocolic excision (CME) and D3 lymph node dissection.

**Fig. 3:**
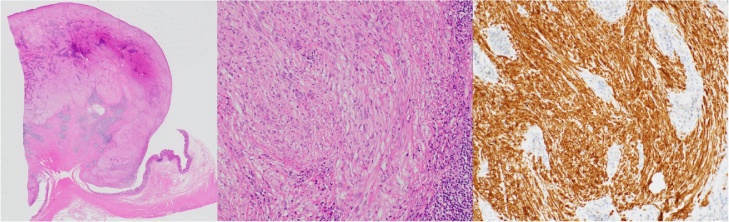
Microscopic pathological findings. (a) At a low-power field, the tumor was found to originate from the muscle layer. (b) At a high-power field, the spindle-shaped cells with weak eosinophilic endoplasmic spores proliferated with fibroblasts. (c) Immunostaining revealed that the tumor cells were positive for S-100 protein.

Assessment of the surgical specimen revealed a well-circumscribed submucosal tumor-like mass. Pathologically, the tumor originated from the muscularis propria layer (Fig. 3a) and spindle cells with weak eosinophilic endoplasmic spores proliferated along with fibroblasts (Fig. 3b). Immunohistochemical staining revealed that the tumor was positive for S100 protein (Fig. 3c) but negative for CD34, c-kit, and desmin. There was no characteristic nuclear sequence indicative of schwannoma. Based on these findings, the tumor was pathologically diagnosed as neurofibroma.

The postoperative course was uneventful, and the patient was discharged 10 days after surgery. During the outpatient visit, her hemoglobin level was within normal limits. No evidence of recurrence and physical symptoms indicative of neurofibromatosis was observed 3 months after surgery. Thus, the patient will undergo an annual outpatient follow up.

## 3 Discussion

Neurofibroma is a benign nerve sheath tumor of the peripheral nervous system, which comprises a mixture of spindle cells with wavy nuclei and collagen strands as well as Schwann cells, perineural-like cells, and fibroblasts [1,3]. Neurofibromas usually originate from the Meissner’s plexus, Auerbach’s plexus, and serosa [4,5]. In the current case, the tumor originated from the muscularis propria layer, which indicates that it originated from the Auerbach’s plexus. Recently, studies on endoscopic resection of neurofibromas have been reported. However, because there is a type of neurofibroma that originates from the muscularis propria layer, the evaluation of tumor depth before resection is considered important.

Although neurofibromas are typically benign lesions, they reportedly undergo malignant transformation and develop into malignant peripheral nerve sheath tumors (MPNSTs), particularly when associated with large or plexiform lesion [6,7]. The term MPNSTs was coined by the World Health Organization and is defined as any malignant tumor arising from the peripheral nerve, such as malignant schwannoma, malignant neurilemmoma, and neurofibrosarcoma [9]. The annual incidence of MPNSTs is 1 person per million of the general population [10]. MPNSTs that arise from the gastrointestinal tract and metastasize to the lymph nodes have been rarely reported [9,11]. In retrospective single-institution studies, large tumor size (typically >5 cm) is the most consistently determined adverse prognostic factor [12].

Gastrointestinal neurofibromas are known to cause clinical symptoms, including bleeding, palpable mass, obstruction, and intussusception. In this case, iron deficiency anemia was caused by microhemorrhage. We believed that curative resection is recommended for the treatment of symptomatic neurofibroma. In contrast, there is no consensus on the exact surgical indications for tumor size owing to the rarity of neurofibromas.

In this case, a high infiltration of lymphocytes and plasma cells into the tumor was observed, which might have caused lymph node enlargement on preoperative CT. However, no studies have reported regarding regional lymphadenopathy on preoperative CT. Therefore, regional lymphadenopathy is not a characteristic feature of neurofibromas. The diagnosis of neurofibroma may not be obtained via preoperative CT.

Endoscopy is typically used to identify single or multiple polypoid lesions or submucosal masses of variable sizes and distributions [7]. However, no patient has been diagnosed with colonic neurofibroma before surgical or endoscopic resection. Endoscopic biopsies may only obtain minimally diagnostic superficial lesion tissues [13], and most MPNSTs are diagnosed via histological examination after excision [11]. In this case as well as in previous reports, endoscopic biopsy only revealed granulation tissues; thus, surgical resection is required for pathological diagnosis. Neurofibromas should be distinguished from submucosal tumors because only granulation tissues are observed on superficial biopsy and boring biopsy.

For neurofibroma, complete surgical extirpation with clear margins is the primary treatment [5,7]. In the current case, the patient underwent laparoscopic right hemicolectomy with CME and D3 lymph node dissection owing to a non-negligible risk of colon cancer. Jung CY reported a case of recurrent neurofibroma that transformed to MPNSTs [12]. However, in general, solitary neurofibromas have a good prognosis and the occurrence of malignant changes and local recurrence is rare [14]. No study has assessed the importance of lymph node dissection for gastrointestinal neurofibroma or MPNSTs. For colonic cancer, CME and central ligation are considered the standard surgical techniques owing to their oncological safety [15]. For colonic neurofibroma, even for MPNSTs as well as colon cancer, colectomy with CME may be sufficient to achieve a clear margin. However, colectomy with CME can be overly invasive for neurofibroma and an accurate preoperative diagnosis allows for local resection. The development of a preoperative diagnostic technique for colonic neurofibroma can help achieve a successful surgery.

In Japan, one case of colonic neurofibroma resected via laparoscopic surgery was reported [16]. However, oncological disadvantages have not been observed. Laparoscopic colectomy for colon cancer, with short-term benefits and long-term oncological outcomes, has been widely used [17]. Although the long-term outcomes of patients with this condition must be evaluated, laparoscopic surgery for neurofibroma is considered feasible.

NF-1 is an autosomal hereditary syndrome characterized by several signs and symptoms [10], and the diagnosis of NF1 must be based on the presence of at least two of the following diagnostic criteria: ≥6 café-au-lait spots, ≥2 neurofibromas of any type or 1 plexiform neurofibroma, freckles in the axillary or inguinal regions, optic glioma, ≥2 iris hamartomas, bony lesions, or a first-degree relative with NF1 [6]. Isolated intestinal neurofibromas may be the initial sign of NF1 in patients without any other clinical manifestations of the disease [6]. In this case, the patient had no signs of NF1 and will undergo an annual outpatient follow up.

The primary limitation of the present study was the inclusion of only a single case. Therefore, large-scale studies involving patients with colonic neurofibroma but without NF1 must be conducted.

## 4 Conclusion

We performed laparoscopic resection with CME for colonic neurofibroma in a patient without NF1. In many cases, a preoperative diagnosis of colonic neurofibroma is impossible. Moreover, neurofibromas should be distinguished from submucosal tumors when only granulation tissues are observed on superficial biopsy and boring biopsy. Laparoscopic surgery with CME for colonic neurofibromas is considered feasible.

## Declaration of Competing Interest

The authors have no conflict of interest to declare.

## Funding

This study did not receive any specific grant from funding agencies in the pubic, commercial, or not-for-profit sectors.

## Ethical approval

This study was approved by the ethics committee.

## Consent

Written informed consent was obtained from patient and her family for publication of this case report.

## Author contribution

TI, SS and TN performed surgery. TI drafted the manuscript. MK participated in the correction of the work. MS, TT, YE and TK were responsible for perioperative management. KI was responsible for endoscopic examination. SI was responsible for pathological diagnosis.

## Registration of research studies

No research study involved in this case report. Not applicable.

## Guarantor

Toru Imagami.

## Provenance and peer review

Editorially reviewed, not externally peer-reviewed
